# Application of Ultrasound in Primary Vesicoureteral Reflux: From Diagnosis to Follow Up

**DOI:** 10.3390/children12101363

**Published:** 2025-10-09

**Authors:** Marco Pensabene, Benedetto Spataro, Fabio Baldanza, Francesco Grasso, Gregorio Serra, Veronica Notarbartolo, Mario Giuffrè, Giovanni Corsello, Elisa Zambaiti, Maria Rita Di Pace, Maria Sergio

**Affiliations:** 1 Pediatric Surgery Unit, Department for Health Promotion, Mother and Child Care and Medical Specialties “D’Alessandro” PROMISE, University of Palermo, 90127 Palermo, Italymariarita.dipace@unipa.it (M.R.D.P.); 2 Neonatal Intensive Care Unit, Department for Health Promotion, Mother and Child Care and Medical Specialties “D’Alessandro” PROMISE, University of Palermo, 90127 Palermo, Italyveronica.notarbartolo@policlinico.pa.it (V.N.);; 3 UOC Pediatric Surgery, Ospedale Infantile “Regina Margherita”, 10126 Torino, Italy

**Keywords:** ultrasound, vesicoureteral reflux, contrast-enhanced ultrasound, children

## Abstract

Background and Objectives: Primary vesicoureteral reflux (VUR) is a common pediatric urological disorder that can lead to significant renal morbidity if undetected or improperly managed. Ultrasound (US) plays a pivotal role in its assessment, providing a radiation-free tool to prenatal assessment, diagnosis, treatment, and long-term follow-up. This study aims to systematically review the literature on the use of US in pediatric primary VUR, emphasizing its applications in prenatal and postnatal diagnosis, intraoperative guidance, and follow-up monitoring. Methods: A systematic review of the literature was performed on PubMed in accordance with PRISMA guidelines. The research strategy used the following keywords: Ultrasound Vesicoureteral reflux, VUR Ultrasound, and VUR Sonography. A total of 2222 records were initially identified. After screening titles and abstracts for relevance, 2165 studies were excluded because they did not focus on ultrasound procedures, did not specify age limits, were redundant, involved non-homogeneous populations, or were unavailable in full text. Results: Prenatal US enables early identification of urinary tract anomalies suggestive of VUR, facilitating targeted postnatal evaluation. Postnatally, contrast-enhanced voiding ultrasound (CEVUS) offers a non-ionizing method for VUR confirmation or exclusion. Intraoperatively, US improves the accuracy and efficacy of bulking agent placement, potentially enhancing surgical outcomes. In follow-up, US remains essential for both conservatively managed and surgically treated patients, enabling timely detection of complications or recurrence. Conclusions: Ultrasound represents a useful tool in the management of pediatric primary VUR, applicable across all clinical stages, avoiding radiation exposure, and improving surgical effectiveness and follow-up management.

## 1. Introduction

The applications for ultrasound (US) in pediatric urologists’ clinical practice are well known, but its use is increasing in recent years. In fact, US is a valuable, cost-effective, and largely available tool in the evaluation and management of a number of conditions in pediatric urology, from prenatal diagnosis to treatment and follow-up. It is due not only to a wider field of applications but also to progressive technical refinement and to technological improvements that have led to a constant increase in the quality of US images, a reduction in costs, and a progressive miniaturization of devices. Moreover, the use of ultrasound in pediatric patients finds its natural allocation, since it preserves patients from exposure to ionizing radiation, from which genetic damage increases with time, making children particularly vulnerable.

It is particularly indicated in urology, since urine provides an optimal window, allowing useful anatomic detail in most cases. Pediatric urologists, thus, should be confident with the use of US for diagnosis and treatment of different anomalies of the kidneys and urinary tract. The aim of this review is to discover the role of the US in the management of congenital vesicoureteral reflux, a common urological condition in children, characterized by the retrograde flow of urine from the bladder into the upper urinary tract [1].

This phenomenon can lead to recurrent urinary tract infections and potentially serious complications, such as renal scarring and chronic kidney disease. Although this condition is considered as a transient and physiological condition in infants, its early diagnosis and appropriate management could prevent long-term sequelae, such as chronic kidney disease and renal failure [2]. With regard to VUR, the use of US could be helpful from initial diagnosis to follow-up, as well as in the treatment itself, with intraoperative US-assisted procedures, since its use improves the effectiveness of surgery, like endoscopic treatment of reflux. This tool allows the surgeon to closely monitor the upper urinary tract in those cases that are managed conservatively, thereby avoiding the use of more invasive or radiation-based imaging modalities that have traditionally been employed for this purpose [1,2,3,4]. This review aims to provide the reader with an analysis of the use of ultrasonography, specifically in primary VUR. The distinctive feature of this review lies in its comprehensive approach, encompassing the various aspects of the management of patients with VUR, from the prenatal period through to the intraoperative field and follow-up.

## 2. Materials and Methods: Papers Selection

This paper’s research was conducted through the PubMed online database. We included all papers published between January 2015 and December 2024. The research was conducted, according with Supplementary Materials: PRISMA guidelines  [5], inserting the following key-words: “Ultrasound Vesicoureteral reflux” (455 papers); “VUR Ultrasound”(1181 papers); “VUR Sonography”(586). The following filters were used: publication date from 1 January 2015 to 31 December 2024; age: child 0–23 months and child 2–5 years; language: English; species: humans. In order to reduce bias due to heterogeneity and paper selection, the analysis of papers was carried out independently by two authors (MP and BS) and supervised by another author (MS). A specific protocol for the review process was assessed and planned prior to the process. The review has been registered to the specific PROSPERO register (ongoing).

## 3. Results

Taking into account the aforementioned keywords and applying the described limits, 2222 studies were found. Of these, after a selection process, 2165 papers were excluded because they were not focused on ultrasound procedures, did not specify age limits, were redundant, involved non-homogeneous populations or because the full paper was unavailable in English. Finally, 57 papers were included in the review process.

**Figure 1:**
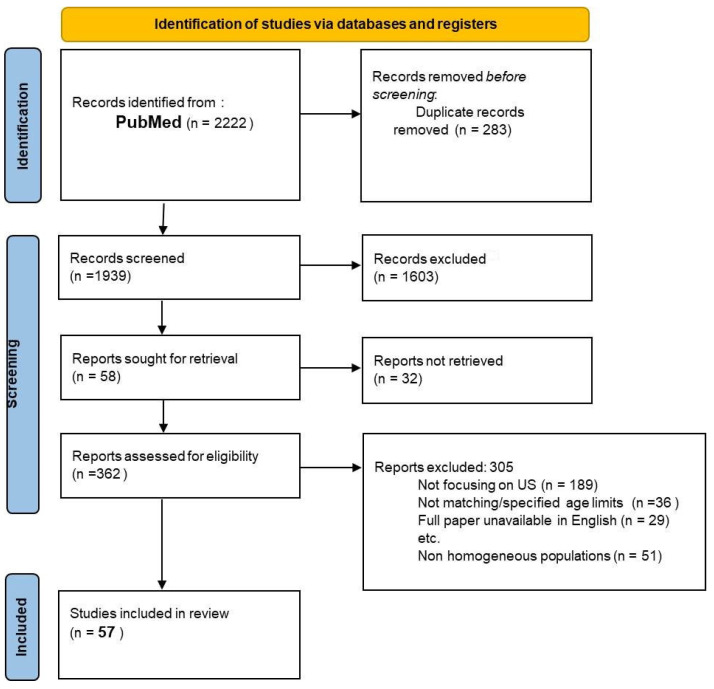
The picture summarizes the paper selection process.

The selection process is summarized in Figure 1.

## 4. Diagnosis of VUR

The diagnosis of VUR could follow a postnatal investigation in patients with prenatal detection of kidney anomaly or dilation or in those experiencing recurrent urinary tract infections (UTIs) in the postnatal period. In both cases, US investigations play a key role in the selection of patients who deserve further investigations and in both confirming or excluding VUR.

Prenatal Ultrasound

Anomalies of the urinary tract may be found in up to 3–5% of prenatal US examinations [6,7,8]. These investigations are usually performed by gynecologists during the routine prenatal assessment, as kidney and bladder visualization is easily performed and is considered a part of a complete fetal US exam [7,9]. Nevertheless, the pediatric urologist should be familiar with using US on the fetal urinary tract, as specialist parental counseling could be required. It should be underlined that the detection and assessment of congenital anomalies of the kidney and urinary tract (CAKUTs) in fetal US is not intended for diagnostic purposes, but it rather aims to identify newborns whose anomalies have the highest risk of complications in postnatal life—such as urinary tract infections and, ultimately, renal failure—where dilation is the initial manifestation. Therefore, it helps determine cases where it is more appropriate to continue with specific diagnostic procedures and those in which a conservative approach could be performed.

In 2014, a multidisciplinary consensus on fetal urinary tract (UT) dilatation underlined the lack of uniformity in defining and grading this condition, with descriptive, quantitative, and semi-quantitative systems available, thus stressing the need for a single grading system that can be used both in the prenatal and postnatal period, in order to promote and facilitate communication between different specialists. The consensus proposed that the concept of hydronephrosis, a kidney-centered point-of-view, should have been replaced by the concept of urinary tract dilatation (UTD), which underlines the importance of the entire urinary tract system, from the pelvis to the bladder, and thus the evaluation not only of the antero/posterior renal pelvis diameter (APRPD) but also of other qualitative parameters like calyceal dilatation, renal parenchymal thickness and appearance, and bladder and ureteral abnormalities [6,7].

The consensus elaborated a risk stratification of patients, in order to select those with low, intermediate, or high risk for detection of CAKUTs and the need for further investigations or surgical intervention. A CAKUT can be detected in 25–50% of antenatal detected UTD cases, and, as a general consideration, the spontaneous resolution rate of UTD is generally lower. The greater the dilatation, the greater the impairment of the other analyzed qualitative parameters [10,11,12,13,14].

It should be emphasized to this point that the direct correlation between the severity of prenatal dilation and the risk of CAKUTs seems to be weaker for vesicoureteral reflux, as this condition can be found in up to 25% of fetuses with renal dilations, regardless of their severity, and in 15% of patients with normal postnatal US [6,7,10,13,15].

Following these considerations, the subsequent topic to be addressed is patient selection—specifically, the identification of newborns with a prenatal suspicion of vesicoureteral reflux (VUR) who may benefit from early diagnosis, even in the absence of a UTI.

In a recent review, Farrugia and Montini extensively explored this topic [16]. The authors report that the presence of ureteral dilatation and abnormalities in the renal prenatal ultrasound appearance are the main indicators for performing postnatal voiding cystourethrography (VCUG). However, the authors underline that the primary aim of VCUG should be to exclude urinary outflow obstruction rather than to diagnose vesicoureteral reflux (VUR) per se.

The authors underline the differences between “congenital VUR” and “symptomatic VUR” diagnosed after UTI occurrence, already reported by Moriquand et al. in 2006 [17]. The first clinical entity, in fact, seems to be bilateral and high-grade in most cases, and it involves males 3 times more often than female patients; moreover, in up to 50% of involved patients, DMSA renal uptake defects are recognized even in those without a UTI, suggesting in utero renal damage.

Nevertheless, one aspect emphasized by the authors is the challenge of reconciling often conflicting findings when comparing patients with a prenatal suspicion of VUR to those diagnosed postnatally following a urinary tract infection (UTI) in two reported prospective studies. Sjöström et al., for instance, reports poorer renal function in the former group compared to the latter, attributing the difference to a higher grade of VUR in the prenatally diagnosed population. In contrast, Mohammadjafari et al. found no significant differences between the two groups, either in terms of renal function or in the incidence of recurrent UTIs [18,19]. According to the European Society of Pediatric Urology Guidelines, in asymptomatic patients with prenatally suspected VUR, only US should be initially performed, despite normal US appearance of kidneys not excluding VUR [20]. The guidelines suggests that the first US should be performed in these patients one week after birth, in order to avoid false negatives images due to physiologic neonatal oliguria, while a second normal US performed within two months of life can exclude VUR in most cases.

Post-natal diagnosis

The continuation of investigations with VCUG could appear straightforward in patients with medium- and high-risk prenatal observed UTD; however, the same cannot be said for those without a prenatal diagnosis or with low-risk prenatal UTD. According to European and American guidelines on pediatric urology, VCUG remains the gold standard for the diagnosis of VUR, even if it is no longer considered a routine exam given the radiological risks associated with the procedure [20].

Indeed, the literature has focused extensively on the selection of patients for whom VCUG should be performed, and many authors have speculated whether US examination could provide sufficient data to successfully select patients who may benefit from VCUG and VUR diagnosis.

In 2016, Gordon et al. found that in patients with “prenatal hydronephrosis”, the presence of two out of four specific postnatal US qualitative parameters (pelvic urothelial thickness >1 mm, hydronephrosis, renal duplication, and renal dysmorphia) increases the sensitivity and specificity of the procedure in predicting high-grade VUR [21].

A subsequent retrospective analysis performed by Visuri et al. in 2018 observed that reduced renal length and ureteral dilation are US predictive parameters of high-grade VUR and thus should be considered an indication for VCUG [22].

Scott-Wang et al., in 2023, developed a machine learning algorithm for selecting patients with dilating VUR on the basis of US UTD and demographic parameters. The authors retrospectively analyzed medical records of infants < 90 days, without a previous UTI, who underwent early ultrasound for prenatal hydronephrosis before positive VCUG. This study aims to standardize the imaging procedures and indication for VCUG for asymptomatic patients with hydronephrotic kidneys [23].

The EAU guidelines report that, after the first UTI, US represents the first-line investigation. In these cases, renal abnormalities are detected in up to 15% of cases, and in 7% of patients, further diagnostic work-up is required [24].

In a recent paper, upper tract dilatation ≥ 7 mm on APRPD has been identified as the most significant independent risk factor for recurrent febrile UTIs in patients with primary VUR on multivariate models [25].

In a retrospective study, Park YW et al. examined abdominal US images of newborns with hydronephrosis. Patients were enrolled in the study group based on the presence of hydronephrosis with a previous UTI or in the control group if the hydronephrosis was without a previous UTI [26].

The authors found that the grade of hydronephrosis was unrelated to VUR, but a significant relationship was observed between ureteral dilatation and VUR in the UTI group (*p* < 0.015), with higher strength in case of high-grade VUR (*p*< 0.005). The authors concluded that the ureteral dilatation should be considered as a helpful parameter to predict high-grade VUR in patients with hydronephrosis experiencing UTIs but not in those without UTIs.

In 2016, You SK et al., focusing on US renal pelvic-wall thickening and pelvic dilatation, concluded that a normal renal/bladder US, with normal DMSA, can exclude high-grade VUR in patients younger than two years [27].

Based on the same concept, some authors, in 2016, demonstrated the link between abnormal US and high-grade VUR. Therefore, they concluded that children with normal US may not require voiding cystourethroscopy [28]

In 2020, Gaither et al. focused on the cost-effectiveness of US screening in patients experiencing UTIs [29]. The authors suggest that, in light of meeting cost-effectiveness criteria, US screening should be performed after the second UTI, rather than after the first, as recommended by guidelines. Moreover, the authors report that this approach seems to significantly reduce unnecessary VCUG.

In order to reduce costs and unnecessary VCUG, Leahy et al. focused on renal–bladder ultrasound (RBUS) timing after the first febrile UTI [30]. The authors found that a delay in RBUS reduces the odds of abnormal dilatation, thus reducing the rate of false positive exams and the need for further screening with VCUG.

Recently, Ishimori et al. reported a retrospective study focusing on 343 patients who underwent renal ultrasound during their first febrile UTI. The authors found that the temporary nephromegaly observed with US in these patients could be considered a predictive factor for UTI recurrence and VUR [31].

In a recent review, Shaik N et al. compared RBUS and DMSA scans to evaluate their accuracy as alternative imaging tools to VCUG in VUR diagnosis [32]. Twenty studies reported data on RBUS, of which 11 focused on high-grade VUR; RBUS showed an overall sensitivity and specificity of 0.44 and 0.78, respectively, in detecting VUR, with slightly higher values of 0.59 and 0.79 in case of high-grade VUR. Nineteen studies focusing on DMSA scans’ effectiveness were included. Overall, values of 0.75 and 0.48 for sensitivity and specificity were found; even in the case of DMSA scans, the diagnostic accuracy increases in case of high-grade VUR, with values of 0.93 and 0.44 for sensitivity and specificity. The authors concluded that neither DMSA nor RBUS could replace VCUG in VUR detection. Thus, despite every attempt at reducing the need for VCUG, it is still considered the gold standard in the diagnosis of VUR, and its use is most suitable in cases in which the definition of anatomical details strongly impacts the diagnosis, as in urethral obstructions or bladder diverticula [20].

Finally, the use of VCUG is recommended in patients with US findings of bilateral high grade, hydronephrosis, duplex kidneys with hydronephrosis, ureterocele, ureteric dilatation, and abnormal bladders according to the EAU guidelines in pediatric urology [20], while in other cases, its use remains optional and subject to the surgeon’s decision.

Nonetheless, in recent years, contrast-enhanced voiding ultrasound (CEVUS) has emerged as a useful imaging alternative to VCUG, with encouraging results in terms of diagnostic effectiveness and, at the same time, saving the patient from exposure to ionizing radiation.

In 2022, Yousefifard M et al. reported a systematic review and meta-analysis on the effectiveness of CEVUS in the diagnosis of VUR [33]. The authors included 36 papers in their extensive review published by 2020, for a total of 2768 children involved. Satisfying results are reported, in terms of sensitivity and specificity of CEVUS: 97% and >93%, respectively, for both first- and second-generation contrast agents. Nevertheless, the authors concluded that, despite a promising technique, CEVUS carries a 3% risk of false negative results.

In 2023, Ye ZLat et al. compared the effectiveness of both techniques, CEVUS vs. VCUG, in preoperative and postoperative follow-up of 159 patients, of whom 78 presented with VUR diagnosis and a total of 60 ureteric units were treated. The authors report that CEVUS offered good performance in preoperative screening for VUR, with values of sensitivity and specificity of >0.83 and >0.97, respectively, accuracy higher than 98%, and positive and negative predictive values of > 95%. Similarly, in the postoperative follow-up, CEVUS sensitivity and specificity exceeded 71%, and its accuracy, positive predictive value, and negative predictive value exceeded 92% [34].

Straus Takahashi M et al., more recently, in 2024, reported their result of a prospective comparison between CEVUS and VCUG. The two procedures were performed consecutively in all 41 involved patients. The authors found similar results, in terms of accuracy and costs [35].

A recent study, published in 2025, evaluated the characteristics of the contrast agent used in both procedures (CEVUS vs. VCUG) and demonstrated that the CEVUS agent is very similar to urine with a lower viscosity compared to the VCUG contrast. The qualities of the contrast agent appear to be important for reproducing the physiological movement and flow of urine in the urinary tract and bladder. Therefore, it appears that it is possible to diagnose a higher number of reflux cases with CEVUS, including those of a higher grade [36].

Some authors have explored patients‘ and parents‘ acceptance of CEVUS compared to VCUG. Back SJ et al. reported that 93–96% of parents identified CEVUS as the preferred modality over conventional VCUG, citing its performance in a more patient-friendly environment, often within facilities already familiar to the child from prior examinations, as well as the opportunity for closer parental presence and involvement during the procedure [37]. Similarly, Seelback J et al. reported that 96% of parents preferred CEVUS over VCUG, and they quietly accept to repeat the procedure if necessary, due to the lack of exposure to ionizing radiation; moreover, CEVUS was described as a less stressful procedure [38].

Overall, CEVUS seems to gain more and more acceptance as a valid diagnostic tool [38,39,40], and recently the European Society of Pediatric Radiology strongly promoted the use of CEVUS, when feasible, limiting VCUG for patients with suspected urethral anomalies [41].

The review study also highlights another positive aspect of CEVUS, namely the possibility of repeating the bladder filling and emptying cycles during the procedure, given the absence of ionizing radiation, which represents a significant risk for the developing body [42,43].

Different CEVUS contrast agents are available [44,45,46].

Despite the procedure being largely standardized, it remains operator-dependent in the absence of ongoing training and practice that ensure sufficient experience [47].

Contrast-enhanced voiding should be performed following five key steps [19]:

1. B-mode US investigation: in supine position, to visualize kidneys and bladder.
2. Bladder catheterization: a sterile gentle procedure to be performed with great care in order to reduce the risk for iatrogenic UTI.
3. Contrast agent preparation: The agent should be prepared according to the manufacturer’s indications, a few minutes before the procedure. The agent is diluted in saline solution and gently agitated. Some authors have suggested the use of pre-warmed solution to be inserted into the bladder, as a rapid change in solution temperature could compromise the agent microbubble stability. The contrast agent is inserted into the bladder via a three-way stopcock linked to the catheter and to the saline solution. A dose of 0.5–1 mL of contrast solution is considered adequate for a single examination.
4. Voiding examination: A complete cycle of bladder filling and emptying should be observed. The scans should start from the bladder in order to visualize the ureters and continue to both kidneys during the filling and emptying phases.

It should be noted that VUR could be a transient phenomenon; thus, multiple complete cycles of bladder filling and emptying may be required for proper VUR detection. The bladder catheter is left in situ and the patient voids around it. To this point, a proper catheter size should be used: a too-large catheter may prevent the patient from voiding during the exam, but a too-small catheter may interfere with the contrast agent (CA) mixing with the intravesical saline, resulting in poor-quality images or in excessive prolongation of the exam, which will become a stressful experience for both the patient and parents, finally compromising the accuracy of the exam itself [48].

**Figure 2:**
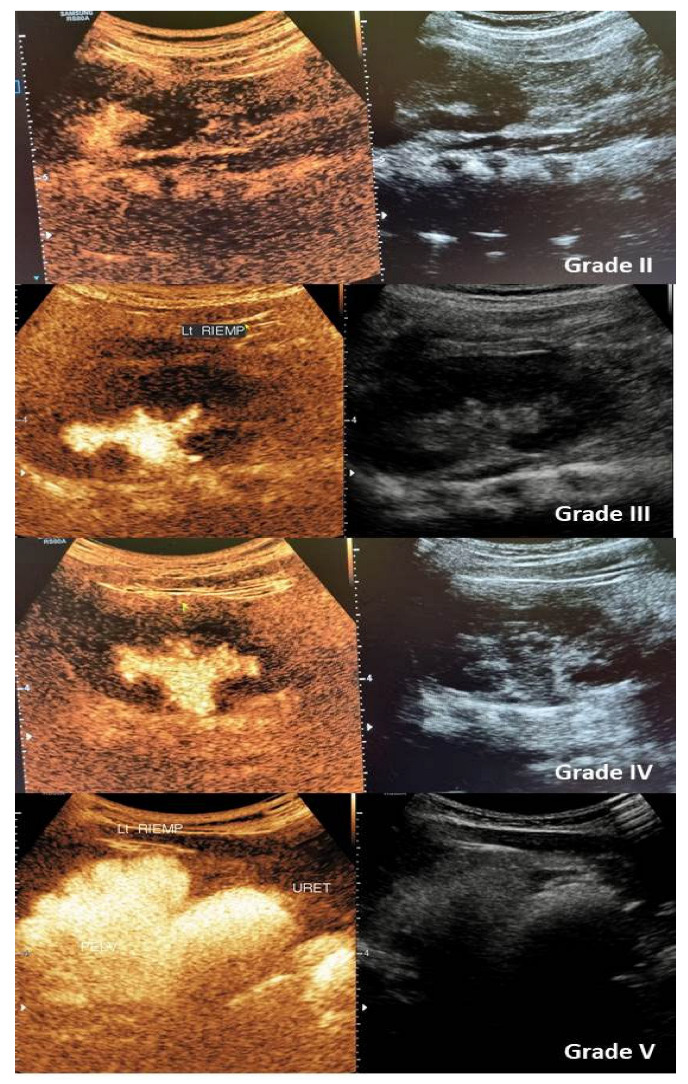
The picture shows the US appearance of grade II-V VUR during CEVUS (**left**) and in B-mode (**right**).

Despite different systems having been proposed for CEVUS classification of VUR, the classic five-degree one, designed based on VCUG findings, remains the most used one in the literature, also when CEVUS is performed. This technique allows a VUR classification similar to the VCUG one, depending on the presence of microbubbles within the dilatation of the ureter and the upper urinary tract (Figure 2) [43,44,45,46,48].

The detection of intrarenal reflux has been investigated by some authors, demonstrating the reliability of CEVUS in detection of this particular condition, occurring in 3–12% of refluxes, especially in higher grades and in children younger than 12 months [48,49]. Intrarenal reflux does not constitute a different grade of reflux but imply a major risk for renal scarring, and consequently, it should be considered an important variant in risk classification and clinical management of VUR [42,49].

## 5. Operative US in VUR

The treatment of VUR is still a matter of debate, with an increasing number of patients being treated conservatively and different and sometimes controversial recommendations regarding surgical treatment available in the literature. Many variables can increase the risk of UTI and persistence of VUR, such as patient’s age, sex, circumcision, presence of lower urinary tract symptoms (LUTSs), and acquired toilet training. These aspects all concur to an unclear definition on what is the best management in patients with VUR. In this complex and variegate scenario, the mininvasive approach with cystoscopic injection of dextranomer/hyaluronic acid copolymer (DX/Ha) has gained more and more importance, proving to be quite as effective as other procedures, with the benefits of minimally invasive surgery in [50]. Nevertheless, there is a lack of objective parameters to predict the effectiveness of the procedure, which may be supposed on the basis of subjective observations during cystoscopy, such as the aspect of the ureteral orifice, the shape of the bulking agent mound, and the hydrodistensibility of the refluxing ureter’s meatus. Thus, the effectiveness of cystoscopic procedures seems to be related to patient selection, surgeon experience, and VUR grade, suggesting difficult standardization of the procedure. The use of intraoperative US can play an important role to overcome this limit, allowing the surgical team to monitor the procedure of injection of DX/Ha during cystoscopy, in order to obtain an effective mound of bulking agent, in a proper submucosal localization [51,52,53]. It has been demonstrated that intraoperative US integration can improve effectiveness of mininvasive procedures for the treatment of intermediate- and high-grade VUR [54].

Three main advantages of US guidance during this procedure can be obtained: 1. It can help the surgeon to better identify the correct submucosal plane where the Dx/HA should be injected and to maintain the cystoscopic needle within it. 2. It follows mound creation until a proper mound height is obtained. 3. It can shorten the surgical time and avoid Dx/HA loss.

**Figure 3:**
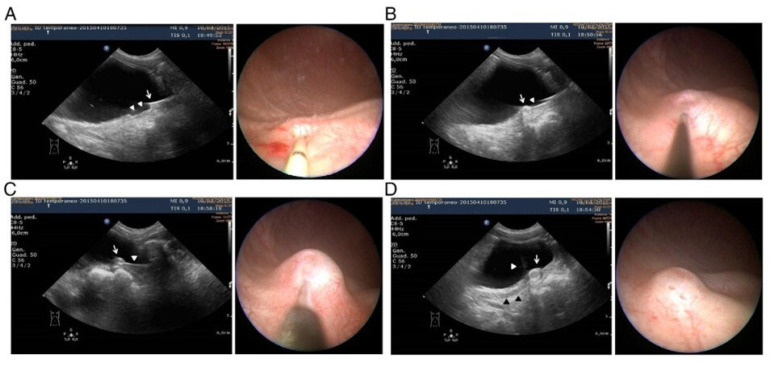
The pictures compare cystoscopic and US appearance of US-guided procedure of bulking agent injection. (**A**) Cistoscopic catheterization of right ureter (arrow cystoscope; arrowheads ureteral catheter); (**B**) recognition of the optimal needle placement, puncture and initial mound formation (arrow initial mound; arrowhead needle); (**C**) injection of Dx/HA and mound increasing (arrow mound; arrowhead needle); (**D**) final implant appearance (arrow mound; white arrowhead ureteral jet; black arrowheads ureteral course).

The site of injection is chosen on the basis of cystoscopic appearance of the ureteral meatus while, at the same time, a bladder US is performed by a second surgeon. The US image should be needle-centered, in sagittal position; an optimal probe position allows the cystoscopic needle to be followed for its whole length. In this phase, it is of crucial importance to maintain the apex of the needle under vision, since it should be kept in the correct submucosal plane when the Dx/HA mound is created. The cystoscopic procedure is then performed and the Dx/HA is injected under direct US guidance until a satisfying mount height is reached (Figure 3).

## 6. Follow-Up

Ultrasound plays a key role in the follow-up of patients with vesicoureteral reflux, both in conservative management and after surgical treatment. Renal morphology, echotexture, renal volume, urinary tract dilation, and bladder emptying are just some of the parameters to be monitored with US. According to data from previous studies, particular importance is given to the diameter and ultrasound appearance of the endovesical mound following endoscopic treatment of reflux with Dx/HA injections. In fact, it has been observed that if a mound height of the bulking agent of less than 10 mm is achieved, an increased recurrence rate of reflux is observed; on the contrary, the persistence of a bulking agent mound with a height greater than 9 mm during follow-up is considered a positive prognostic factor for treatment success [51,52,53,54].

Therefore, ultrasound is an excellent tool for assessing the resorption of the injected agent over time [53].

A 2023 review by Zhao Lan Ye et al. shows that CEVUS has comparable results, in terms of sensitivity and specificity, to VCUG in the 6–12–18-month follow-up of patients undergoing surgery for VUR. A total of 159 children with UTIs were screened with CeVUS and VCUG from December 2018 to December 2020, of whom 78 were diagnosed with VUR. A total of 60 ureteral units underwent surgery. Cevus and VCUG were repeated 6, 12, and 18 months after surgery to evaluate postoperative outcomes. The diagnostic results of both CeVUS and VCUG showed high concordance, with a kappa value of 0.966 (*p* < 0.001) suggesting that CeVUS could offer comparable diagnostic sensitivity to VCUG [34].

A retrospective Israeli study showed the importance of US follow-up in patients who underwent endoscopic injection for VUR correction. The author observed that the postoperative US follow-up showed evidence of late stenosis of the ureterovesical junction, requiring open reimplantation in all reported patients, underlining the central role of the ultrasound evaluation during the follow-up of patients treated with endoscopic injection of bulking agents [55].

## 7. Conclusions

In conclusion, US applications in the management of pediatric primary VUR are wide. Its use provide a safe and effective tool for the management of primary VUR, in the selection of those fetuses who may require further postnatal investigations, and in radiation-free and proper VUR diagnosis and grading. US-assisted procedures offer the surgeon an important tool to improve the effectiveness of bulking agent injection, and in the postoperative evaluation, US allows the surgeon to safely monitor the postoperative condition and detect VUR recurrence early.

We would like to highlight that conventional ultrasound, integrated with contrast-enhanced techniques, by assessing specific parameters such as renal and urinary tract morphovolumetry, echotexture, and echogenicity, allows for the evaluation of the natural history of the disease and the identification of targeted treatments aimed at preventing further impairment of renal function. Moreover, it is important to emphasize that this diagnostic tool is free from ionizing radiation and can therefore be easily repeated.

The use of US should be familiar to pediatric surgeons and urologists, since its applications are expected to extend to other fields in clinical practice and in the operating room, given the progressive improvement of devices.

## Data Availability

No new data were created or analyzed in this study. Data sharing is not applicable to this article.

## Supplementary Materials

The following supporting information can be downloaded at: https://www.mdpi.com/article/10.3390/children12101363/s1, NA PRISMA 2020 Checklist.

## Abbreviations

The following abbreviations are used in this manuscript:

VUR	Vesicoureteral reflux
US	Ultrasound
CEVUS	Contrast-enhanced voiding ultrasound
PRISMA	Preferred Reporting Items for Systematic reviews and Meta-Analyses
UTI	Urinary tract infections
CAKUT	Congenital anomalies of the kidney and urinary tract
UT	Urinary tract
UTD	Urinary tract dilatation
APRPD	Antero/posterior renal pelvis diameter
VCUG	Voiding cystourethrography
DMSA	Dimercaptosuccinic acid
RBUS	Renal–bladder ultrasound
LUTS	Lower urinary tract symptoms
DX/Ha	Dextranomer/Hyaluronic acid Copolymer
